# Comparative Study of the Nutritional, Phytochemical, Sensory Characteristics and Glycemic Response of Cookies Enriched with Lupin Sprout Flour and Lupin Green Sprout

**DOI:** 10.3390/foods13050656

**Published:** 2024-02-21

**Authors:** Loredana Plustea, Sylvestre Dossa, Christine Dragomir, Ileana Cocan, Monica Negrea, Diana Obistioiu, Mariana-Atena Poiana, Daniela Voica, Adina Berbecea, Ersilia Alexa

**Affiliations:** 1 Faculty of Food Engineering, University of Life Sciences “King Mihai I” from Timisoara, Aradului Street No 119, 300645 Timisoara, Romania; loredanapaven@yahoo.com (L.P.); sylvestredossa04@gmail.com (S.D.); christine.dragomir98@gmail.com (C.D.); ileanacocan@usvt.ro (I.C.); monicanegrea@usvt.ro (M.N.); marianapoiana@usvt.ro (M.-A.P.); 2 Faculty of Agriculture, University of Life Sciences “King Mihai I” from Timisoara, Aradului Street No 119, 300645 Timisoara, Romania; dianaobistioiu@usvt.ro (D.O.); adina_berbecea@usvt-tm.ro (A.B.); 3 Romanian Association of Milling and Bakery (ROMPAN), Calea Plevnei nr. 145, București, Sector 6, 060012 Bucharest, Romania; tehnic@rompan.ro

**Keywords:** nutritional, phytochemical, sensory analysis, cookies, lupin, sprout, glycemic index

## Abstract

**Featured Application:**

**The paper has application potential in the bakery and related industries, considering that the proposed solutions offer improved products in terms of the nutritional and functional points of view.**

**Abstract:**

This study aimed to compare the nutritional, phytochemical, and sensory characteristics of wheat flour (WF) cookies enriched with different proportions of lupin sprout flour (LSF) and those with different proportions of lupin green sprout (LGS). To achieve this, a control cookie (CC); three cookies with 10%, 20%, and 30% of LSF, respectively, CLSF1, CLSF2, and CLSF3; and three other cookies (CLGS1, CLGS2, and CLGS3) with 10%, 20%, and 30%, respectively, were produced. The proximate composition of each cookie was analyzed using AOAC methods. Also, the measurements of the total polyphenol content, antioxidant activity, individual polyphenols, glycemic index, and a sensory analysis were carried out using recent and accurate methods. The contribution of the main nutrients from 100 g of product to the required daily dose was also calculated. Data analysis revealed that cookies with LSF were richer than cookies with LGS in protein, fat, and energy values. CLGS3 was 35.12%, 1.45%, and 5.0% lower in protein, fat, and energy content than CLSF3, respectively. On the other hand, CLSF3 was lower than CLGS3, with 48.2% and 12.4% in moisture and mineral substances, respectively. Both cookies were lower in carbohydrates than the CC (65.20 g/100 g). Still on the subject of micro- and macronutrients, cookies with LSF were richer than those with LGS in all the minerals analyzed. The study also revealed improvements in phytochemical properties, such as total and individual polyphenols and antioxidant activity with the percentage of lupin sprout flour addition. The sensory analysis revealed that, for LSF and LGS cookies, the 10% samples were the most appreciated by consumers, irrespective of the sensory attributes studied. The glycemic index of the CLSF2 product was lower compared to the CC. This study shows that the LSF cookies have better nutritional, phytochemical, and sensory values than the LGS cookies. LSF is, therefore, better suited than LGS to the enrichment of bakery products in general and cookies in particular. The paper provides significant information to estimate the contribution of the consumption of functional products based on lupin sprouts to the required daily dose of food nutrients and the impact on the glycemic index of fortified products.

## 1. Introduction

The seeds of various plant species can become, through germination, valuable food or medicine due to their content of active principles used for the treatment of certain diseases. Gemotherapy (“gemmae”—plant bud, “therapea”—treatment) is the youngest of the biotherapies; it is an innovative example of a new and revolutionary way of conceiving phytotherapy. This branch of phytotherapy involves using embryonic tissues of plants, trees, or shrubs (called meristems), shoots, roots, the bark of young branches, sap, seeds, and sprouts due to the content of active principles that are valuable to health [1].

Sprouts are natural products obtained by germinating edible seeds, not chemically treated, under strict hygiene conditions, marketed at the stage of sprouted seeds. Sprouts are an irreplaceable ingredient for those who want a long and healthy life. These germinated seeds are very easy to introduce into the diet; they can be added to salads or used as a garnish for food. In addition to their important nutritional intake, sprouts are also very easy to assimilate by the human body, which consumes a minimum amount of energy to digest them [2].

The germination process initially involves the absorption of water when seed hydration takes place, followed by the activation of metabolism, growth respiration, and protein synthesis [3]. During germination processes, the degradation of the main macronutrients (carbohydrates, proteins, and fatty acids) occurs with the formation of simple sugars, free amino acids, and organic acids [4].

Food technologies aim to use cereal sprouts in easy-to-administer forms that preserve as many of their special properties as live food, favoring the preservation of enzymes and phytohormones, substances very sensitive to high-temperature changes in technological processes.

Initially, the use of germinated seeds mainly focused on cereals, such as wheat, barley, and oats [5,6,7]. Then, the use of plants in the sprout form was extended to other species, obtaining germinated legumes (peas, chickpeas [8], soybeans [9], and lentils [10]), oleaginous (sunflower and pumpkin), as well as the sprouts of certain medicinal plants, fenugreek being the most representative in this sense. More than 40 types of germinated plants are currently used for various therapies. Beyond substances with well-known effects, such as vitamins, mineral salts, and proteins, the chemical composition of sprouts of different species is still incompletely elucidated [11]. Manufacturers of natural medicines have a wide field of research to discover more and more therapeutic properties of these parts of plants that contain numerous substances with spectacular effects. Depending on the species and variety, the sprouting process releases significant amounts of beneficial nutrients, vitamins, and enzymes [12].

The quality of proteins from seeds or grains is improved when they are sprouted. Sprouts can have 100-times more enzymes than raw fruits, and vegetables and have health benefits due to the antioxidant, antimicrobial, and antiproliferative effects [13]. Legume sprouts are a good source of nutrients and active principles and are used to obtain functional, dietary, [14], or prebiotic foods [15].

Sprouts can slow down or even reverse, to a certain extent, the body’s aging process; there are more and more cases of older people regaining, through the constant consumption of germinated plants, some of the qualities specific to maturity or youth [16]. Different researchers claim that this regenerative activity is due to antioxidants, the wide range of enzymes, and antioxidant vitamins A, C, and E. These prevent the oxidation of fats in the blood, thus inhibiting the formation of free radicals. The lipid content of cereal germs, provided by triglycerides that contain linolenic and linolenic acids in significant quantities, as well as the high content of phospholipids, explains their high therapeutic action. Cereal sprouts are also rich in phytosterols, compounds that have an anticholesterolemic effect, and have a high content of tocopherols, active compounds for preventing cardiovascular diseases. Also, antioxidant, antidiabetic, and anticancer effects were reported [4].

Regarding the baking industry, wheat sprouts are used due to their cost and availability, but legume sprouts are an important source of nutrients for enriching flour products [17].

This paper’s aim was to study the cookies produced with lupin sprout flour (LSF) and lupin green sprouts (LGSs) replaced with wheat flour at different percentages, from nutritional, sensory, and phytochemical points of view, in order to use the flours as a sustainable food in a healthy diet. The glycemic index (GI) was determined through in vivo tests for the cookies that proved to have maximum contents in active principles. Also, the contribution of 100 g products to the daily intake of principal nutrients was calculated. Several studies reported the use of sprouts as ingredients in functional foods, to improve the rheological properties of dough in bakery products, or antioxidant activity [18,19,20,21,22,23,24,25,26,27]. However, according to our knowledge, no studies have reported the contribution of the consumption of functional products based on lupin sprouts to the required daily dose of food nutrients and the impact on the glycemic index of fortified products.

## 2. Materials and Methods

### 2.1. Ingredients and Cookie Production

#### 2.1.1. Obtaining Sprouts and Preparing Composite Flours

Lupin sprouts were obtained by weighing 450 g of lupin seeds and soaking them in water at 32–33 °C for 12 h [28]. During soaking, seed moisture increased initially from 10–14% to 42–45% when germination was triggered. To ensure an optimal humidity for the grain germination of 43–48%, a directed soaking was performed. Moisture values of 46–47% ensured the optimal transfer of nutrient components from the endosperm to the germ, enhancing the enzyme synthesis and hydrolysis of macromolecules, especially starch and protein. In general, water absorption was the maximum in the first 8 h, the speed gradually reducing until it reached a degree of saturation [29]. The lupin seeds were then placed in a tray containing absorbent cotton. The tray containing the lupin seeds was placed in a bright room at a constant temperature (22 °C). The seeds began to germinate from day 2 to day 14. They were watered daily to maintain a constant humidity level and, after 14 days, the lupin sprouts were harvested.

**Figure 1:**
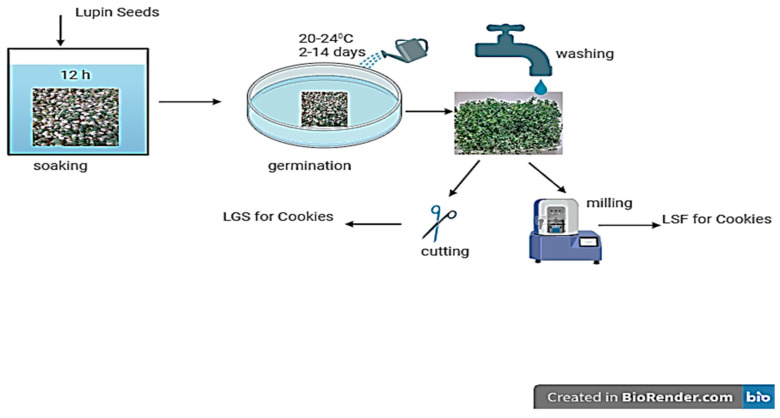
The technological process for obtaining lupin sprouts. Figure created with BioRender.com, accessed on 23 December 2023.

Two types of cookies were produced: cookies with lupin sprout flour (LSF) and cookies with lupin green sprout (LGS) with different proportions of lupin. For the cookies made with LGS, the green, fresh sprouts were washed, coarsely shredded using a knife, and weighed before being introduced directly into the recipe. For cookies with LSF, after harvesting, the green lupin sprouts were dried in a thermostat at 60 degrees for 4 days. After this stage, the dried sprouts were ground in a mill to obtain lupin sprout flour (Figure 1). The flour obtained was then used in combination with wheat flour to create a composite flour. The substitution level was chosen at 10–30% because, according to our previous studies, a higher addition of lupin flour, including 30%, worsens the rheological properties of flour [18]. Six types of composite flours were produced: LSF1 (10% LSF and 90% WF); LSF2 (20% LSF and 80% WF), LSF3 (30% LSF and 70% WF), LGS1 (10% LGS and 90% WF), LGS2 (20% LGS and 80% WF), and LGS3 (30% LGS and 70% WF). Wheat flour (WF) (white type) was purchased from the Auchan supermarket in Timisoara, Romania.

#### 2.1.2. Cookie Preparation

**Figure 2:**
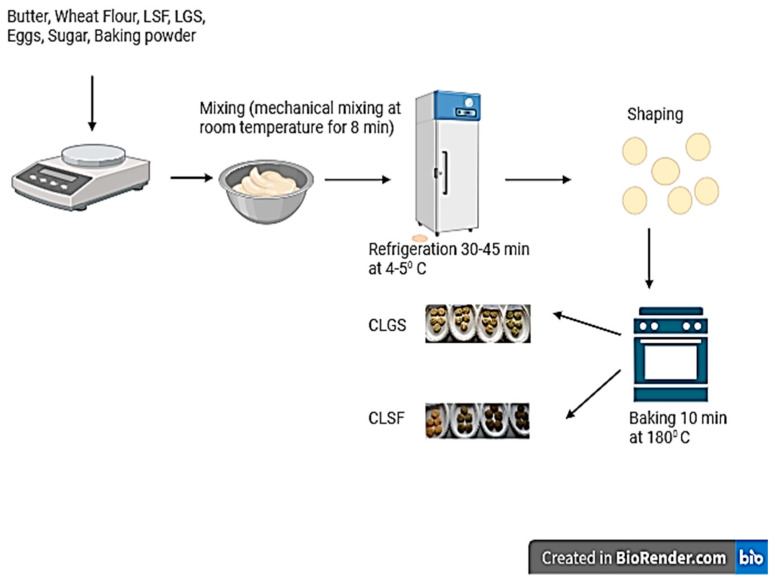
The technological flowchart for obtaining cookies with green lupin sprouts and lupin sprout flour. Figure created with BioRender.com, accessed on 23 December 2023.

**Figure 3:**
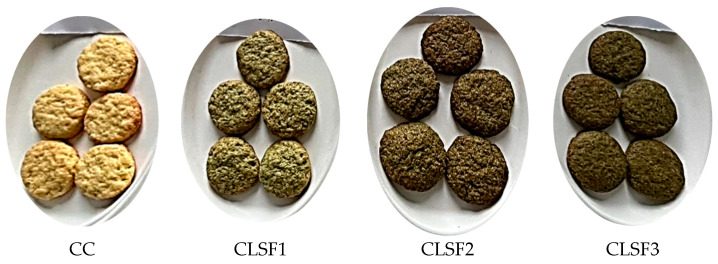
Cookies made with wheat flour (WF) enriched with different proportions of lupin sprout flour (LSF).

**Figure 4:**
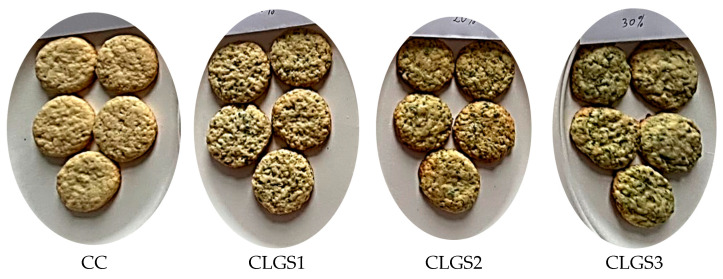
Cookies made with wheat flour (WF) enriched with different proportions lupin green sprout (LGS).

The method proposed by Chopra et al. (2014) with some modifications was used for the cookies’ preparation [30]. The other ingredients (sugar, baking powder, eggs, and butter) were purchased at Lidl and Kaufland supermarket in Timisoara (Romania). Table 1 lists the different amounts of ingredients used in each type of cookie. A total of 1 control cookie (CC—control cookie with 100% WF), 3 cookies with different amounts of lupin sprout flour (CLSF1—cookie with 10% LSF and 90% WF, CLSF2—cookie with 20% LSF and 80% WF, and CLSF3—cookie with 30% LSF and 70% WF), and 3 other types of cookies, but this time with lupin green sprouts (CLGS1—cookie with 10% LGS and 90% WF; CLGS2—cookie with 20% LGS and 80% WF; and CLGS3—cookie with 30% LGS and 70% WF) were made. They were prepared by incorporating LGS or LSF (as appropriate) at 10, 20, and 30% concentrations in wheat flour-type 650 (see Section 2.1.1). Sugar, baking powder, eggs, and butter were added to each previously obtained mixture. The resulting dough was placed in a bowl and cooled (between 4 and 5 °C) for 30 to 45 min. After cooling, the dough was rolled out on a table to a thickness of 1 cm and cut into circles. The cutouts were then placed on a baking tray and baked in a convective oven (Esmach, Venice, Italy) at 180 °C for 10 min. After baking, the cookies were cooled and packed (Figure 2). Figure 3 and Figure 4 show the various cookies produced as part of this study.

### 2.2. Determining the Proximate Composition of Lupin Sprout Cookies

The proximate compositions of the control and composite cookies was determined using the AOAC (2006) method guidelines [31]. AOAC methods were used to determine fat (954.02) and ash (942.05) contents, while methods no. 925.10 and no. 925.36 were used to determine moisture and protein contents, respectively. The total carbohydrate content (%) was calculated as the difference between 100 and the sum of the following fractions: proteins, fats, mineral substances, and moisture.

Macro- and microelement content (mg/kg) were determined using the atomic absorption spectrophotometric method described by Plustea et al., (2022) [18].

### 2.3. Daily Intakes (Dis) of Lupin Sprout Cookies

Daily intakes (Dis) were calculated by relating the values obtained to the required reference intake of an average adult per day, i.e., 50 g of protein, 70 g of fat, 260 g of carbohydrate according to Equations (1)–(3):

(1) $${DIs\;}_{protein}=\frac{p\cdot 100}{50}\;$$

(2) $${DIs\;}_{lipid}=\frac{l\cdot 100}{70}$$

(3) $${DIs\;}_{carbohydrates}=\frac{c\cdot 100}{260}\;$$

where:

*Dis*—daily intakes (%);
*p*—protein content per 100 g sample;
*l*—lipid content per 100 g sample;
*c*—carbohydrate content per 100 g sample.

### 2.4. Phytochemical Profile of Lupin Sprout Cookies

A total of 1 g of each cookie sample was ground using a Retsch laboratory miller (Dusseldorf, Germany) and weighed into lidded containers. To this, 10 mL of 70% (*v*/*v*) ethanol (Chimreactiv, Bucharest, Romania) was added. After 30 min of stirring with a magnetic stirrer (IDL, Freising, Germany), the mixture was filtered through Whatman N°1 filter paper [32].

#### 2.4.1. Total Phenolic Content (TPC)

The method used for measuring the TPC content was the Folin–Ciocalteu method [33]. In this method, to 1 mL of extract, 1.25 mL of Folin–Ciocalteu reagent (Sigma-Aldrich; Merck KgaA, St. Louis, MA, USA) diluted to 1:10 with water was added, and the sample was incubated at 22 °C. After 5 min, to the sample was added 1 mL of Na_2_CO_3_ 60 g/L, and the sample was heated at 50 °C. After 30 min, the absorbance of extracts was measured at 750 nm (Specord 205; Analytik Jena, Bucharest, Romania). Standard gallic acid (GA) solutions (Sigma-Aldrich; Merck KgaA) at concentrations ranging from 0.1 to 1.0 mM of gallic acid equivalents (GAEs)/Ml were used for the calibration curve. The regression equation was y = 1.92x − 0.10 and the coefficient of correlation was R^2^ = 0.9980. The results are expressed in mg GAE/g. All experiments were performed in triplicate.

#### 2.4.2. Antioxidant Capacity

The method used was specified in the study of Floares et al., (2023) [34]. A total of 1 mL of extract and 2.5 mL of a DPPH (1,1-diphenyl-2-picrylhydrazyl) 0.03 mM solution were shaken. After 30 min at room temperature, the absorbance was read at 518 nm (Specord 205; Analytik Jena AG, Jena, Germany). The results are presented in µgTROLOX/mL. The sample was analyzed in triplicate.

To assess the antioxidant activity of fortified cookies by the ferric-reducing antioxidant power (FRAP) method, 0.5 mL of extracts were mixed with 2.5 mL of the FRAP solution [34]. After 30 min at 37 °C, the absorbance of samples was measured at 590 nm (Specord 205; Analytik Jena). A standard solution of FeSO_4_·7H_2_O (Sigma-Aldrich; Merck KgaA) was used for the calibration curve. The regression equation was y = 2.3559x − 0.0189, R2 = 0.9986, and the coefficient of correlation was R2 = 0.9980. The results are expressed as µgFe^2+/^g. All experiments were performed in triplicate.

#### 2.4.3. Assessment of Individual Polyphenols

Polyphenols were separated and identified by LC-MS (Shimadzu 2010 EV, Kyoto, Japan) according to a technique described by Duca et al. [35]. The separation conditions were a Nucleodur CE 150/2 C18 Gravity SB 150 mm × 2.0 mm column (Macherey-Nagel GmbH & Co. KG, Duren, Germany) and a flow rate of 0.2 mL/min. Gradient elution: acetonitrile pH 3; aqueous formic acid solution: pH 3: (i) 0.01–20 min—5% acetonitrile, (ii) 20.01–50 min—5–40%, (iii) 50–55 min—40–95%, and (iv) in the range of 55–60 min—95% acetonitrile. Calibration curves were created in the range of 20–50 μg/mL. The measurements were performed in triplicate (limit of detection: 0.4–0.5 µg/mL; limit of quantification: 0.6–0.7 µg/mL). Standard solutions were prepared from 1000 µg/mL standard solutions of epicatechin, caffeic acid, rutin, rosmarinic acid, quercetin, gallic, coumaric, ferulic, and beta-resorcylic.

### 2.5. Physical Evaluation of Lupin Sprout Cookies

The diameter, thickness, and spread ration of the cookies were measured using the method described by Arun K.B. et al., (2015) [36]. The diameters of 6 cookies from each assortment were measured using a scale; then, the cookies were rotated and re-measured again and the average of the two measurements was calculated. Using a caliper, the thickness was measured at two distinct heights and the average was considered. Spread ratios were calculated as a ratio of the diameter to the thickness.

### 2.6. Sensory Analysis of Lupin Sprout Cookies

The sensory analysis was carried out according to the methods of Iannario, M. et al., (2012) [37] and Pestorić et al., (2017) [38], in compliance with the laboratory’s ethical guidelines as well as the written consent signed by each evaluator according to the European Union guidelines on ethics and food-related research [39]. The sensory evaluation was performed by panelists, semi-trained employees, and students of the University of Life Science Timisoara, Romania. The panel was composed of 15 evaluators (7 men and 8 women), who were non-smokers aged between 19 and 46 years, with no known cases of allergies, especially to gluten or food intolerances. The panelists were trained for attribute identification before the evaluation. The samples were presented individually in cardboard dishes with three-digit characters to each assessor. It should be noted that the panelists rinsed their mouths with plain water after testing each sample. The coded samples’ sensory characteristics (appearance, flavor, texture, taste, and overall acceptability) were rated according to their degree of likeness using a five-point hedonic scale [38].

The panelists presented scores of 1–5 for each attribute analyzed as follows:

Appearance: 1—unattractive, 2—slightly unattractive, 3—moderate, 4—attractive, and 5—very attractive.
Flavor: 1—dislike, 2—neither like nor dislike, 3—like slightly, 4—like moderately, and 5—like very much.
Texture: 1—very poor, 2—poor, 3—fair, 4—good, and 5—very good.
Taste: 1—very poor, 2—poor, 3—fair, 4—good, and 5—very good.
Overall acceptance: 1—dislike, 2—neither like nor dislike, 3—like slightly, 4—like moderately, and 5—like very much.

### 2.7. Determination of the Glycemic Index (GI) of Lupin Sprout Cookies

The study was conducted in accordance with the Declaration of Helsinki and Good Clinical Practice Regulation (CPMP/ICH/135/95) and the European regulatory requirements (Directive 75/78/EC). The study protocol was approved by the University of Life Sciences Bioethics Committee, “King Mihai I” from Timisoara (202/10.03.2023). Written informed consent was obtained from all participants, with oral information provided and the study protocol written. A panel composed of 15 volunteer evaluators (7 men and 8 women), who were non-smokers, aged between 19 and 46 years, with no known cases of allergies, especially to gluten or food intolerances, were selected.

The testing was carried out on healthy volunteers after signing the Informed consent forms, following the determination of the glycemic index relative to 50 g of glucose pulvis.

The objectives of the research activity were: (i) establishing the daily amount to determine the functional effect; (ii) establishing tolerance and palatability; and (iii) proof of functional effect through markers.

#### 2.7.1. Safety Objectives

All the study participants were evaluated for safety. The safety review included assessments of adverse reactions and laboratory values. Adverse reactions were summarized and presented using the link to the foods included in the study and adverse reactions to a specific herb or bioactive compound.

#### 2.7.2. The Population Included in the Study

Healthy male or female volunteers aged at least 18 years participated in the present nutritional study after signing an informed consent form.

Exclusion criteria were: (i) personal and family histories, including any type of cancer or degenerative disease; (ii) irritable bowel syndrome, inflammatory bowel diseases, or other diseases that cause diarrhea; (iii) active gastric or duodenal ulcers; (iv) abnormal ECG clinic; (v) abnormal lung X-ray; (vi) abnormal abdominal ultrasound; (vii) participation in any other clinical trial in the last month; (viii) deterioration of hematological, hepatic, or renal functions; and (ix) known hypersensitivity or allergy to any of the product’s components.

People who suffered from a syndrome or had certain deficiencies were excluded from the study.

#### 2.7.3. Description of Testing on Healthy Volunteers to Determine the Glycemic Index of the Lupin Sprout Cookies

The study consisted of consuming each functional cookie in a meal equivalent to 50 g of carbohydrates from the product, compared to the effect of 50 g of glucose on blood sugar. Participation in this study was voluntary; the participants had the right to withdraw from the study at any time without providing reasons for their decision and without any prejudice in terms of medical assistance.

The analysis was performed in the morning in a fasting state, after a minimum of 10 h of food rest. The blood glucose level was measured in the capillary blood obtained by pricking using a lancet and reading it with a glucometer. Blood sugar levels were measured in 5 periods (0, 30, 60, 120, and 180 min). The trapezoidal rule calculated the glycemic response or incremental area under the curve (IAUC) for each reference food tested [40]. The glycemic index (GI) was calculated according to Formula (5) [41,42].

GI = (IAUC of the test food/the IAUC of reference food) × 100 (4)

### 2.8. Statistical Analysis

The results represent an average of three independent determinations and are expressed as the mean ± standard deviation (SD). A one-way analysis of variance (ANOVA) was performed, and differences were considered statistically significant with a probability less than 0.05 (*p* < 0.05). The software used was Microsoft Excel 365 (Version 2208, Redmond, WA, USA).

## 3. Results and Discussion

### 3.1. Nutritional Characteristics and Daily Intakes (Dis) of Composite Cookies

To establish the proximate composition of the different cookies produced, their contents in terms of moisture, minerals, proteins, lipids, and carbohydrates, as well as the energy value each provided, were determined. The results are presented in Table 2.

The data analysis in Table 2 shows that cookies with different proportions of sprout flour or green lupin sprout flour are richer in minerals, proteins, and lipids than the control cookie. However, it should be noted that the control cookie contains more water than the LSF cookies, but no more than the LGS biscuits. The CC is richer in carbohydrates than both types of cookies with lupin. It provides less energy than the LSF cookies, but more than the LGS biscuits.

In terms of the moisture content, cookies with LSF contained less water than the CC. Humidity in the LGS cookie was 58.5% compared to the LSF cookie, where a lower water content was determined (10.2%). In addition, the moisture content decreased as the LSF increased in the cookie’s composition. Similar results were reported in our previous study when lupin flour was used in a composite flour for bread recipes [18]. On the other hand, in the studies of Alomari and Abdul-Hussain (2013) [43], although lupin flour contained less water than wheat flour, the breads produced with different percentages of lupin were richer in moisture than the control bread. Several aspects, such as the production method and the ingredients used, can explain this difference. It was observed that lupin flour, at a 5% substitution level, increased the stability and tolerance index of the dough [44]. In our study, CLSF1, CLSF2, and CLSF3 resulted in 6.7 ± 0.2, 6.1 ± 0.1, and 5.8 ± 0.2%, respectively, compared with 6.7 ± 0.2% for the control sample. In contrast to the cookies with LSF, those with LGS had the highest moisture contents, ranging from 7.5 ± 0.4 to 11.3 ± 0.4%. This is explained by the fact that LGS contains more water than LSF.

Both types of cookies with lupin contained more minerals than the CC. This is explained by the fact that lupin is richer in mineral substances than wheat flour [18,43,45]. We also found that mineral substances were more abundant as lupin was increased in the LSF and LGS cookies, in agreement with the findings reported by other authors [18,43,45]. The mineral content increased with the addition of LSF, containing 5.7% more than LGS (1.0%).

Significant differences (*p* < 0.05) were observed for the mineral content in the case of all the experimental variants of CLGS, but not between the variants with 20 and 30% of lupin flour (CLSF). The variant with a 10% addition of flour or green lupin sprouts did not vary significantly in its mineral content compared to the control variant (CC). In fact, CLSF1, CLSF2, and CLSF3 contained 1.0 ± 0.1, 1.3 ± 0.0, and 1.3 ± 0.0%, respectively, while CLGS1, CLGS2, and CLGS3 contained 1.1 ± 0.1, 1.3 ± 0.1, and 1.5 ± 0.1% mineral substances, respectively. It can, therefore, be deduced that the transformation of LGS into LSF has no impact on the mineral composition.

The increase in the mineral content (zinc, calcium, magnesium, potassium, and iron) during the germination of legumes compared to non-germinated seeds and grains was previously reported [46,47]. The authors explained this phenomenon occurred due to the breakdown of phytates by the action of the enzyme phytase. It was reported that the increase in the total mineral content from 2.71 to 3.25%, Fe from 57.00 to 63.00 mg/kg, Cu from 10.00 to 17.00 mg/kg, and Zn 28.00–32.00 mg/kg [47] occurred during the germination of the legume seeds.

In terms of the protein, the CC contained less than the others. This is explained by the fact that lupin is richer in protein than wheat flour [18,43,48,49]. The protein levels rose from 7.9 ± 0.1% for the CC, 8.6 ± 0.1% for CLGS3, and 13.3 ± 0.1% for CLSF3. Protein levels increased with the amount of LSF and LGS, which is in agreement with other studies that reported an increase in the protein content in the composite lupin flour from 14.67%, for the 10% lupin flour addition, to 52.92%, in the case of the 30% lupin flour addition to wheat flour. Bread with 10% lupin flour showed an increase of 13.10% of protein compared with the control bread, while a 20% addition of lupin flour to wheat bread lead to a 35.02% increase in protein content; a 58.75% increase was observed for a fortified percentage of 30% with lupin flour [18]. The protein content of lupin flour is superior to that of wheat flour, and the addition of sprouts, both green and in the form of flour, leads to an improvement in the protein profile of cookies. The increase in protein content relative to the control varied in the range of 14.42–67.99% for CLSF and 0.58–9.063% for CLGS (Table 2).

During germination, storage proteins are destroyed by proteases and provide free amino acids [50]. Previous studies reported an increase in protein content in the range of 2–10% during germination, depending on the type of seed [3]. The increase in the protein content can be attributed to the breakdown of fats and carbohydrates and the synthesis of proteins and free amino acids [50]. The proteins from cereal sprouts, compared to the proteins of the cereals from which they come, contain amino acids with a balanced composition. This aspect is important for the body’s defensive system by favoring the formation of antibodies while also favoring the individual’s brain activity and physical performance. Proteins have an effect on the metabolic balancing of diabetic patients as well as on the nutrition of children where an additional intake of proteins is necessary. Also, proteins from cereal sprouts, including wheat, are low in gluten and can be used to nourish children with a gluten intolerance [7].

Like the protein content, the lipid content of cookies containing lupin was higher than that of the CC. Moreover, cookies with LSF were richer in lipids than those with LGS. CLSF1, CLSF2, and CLSF3 contained 22.1 ± 0.2, 22.3 ± 0.2, and 22.4 ± 0.1%, respectively, while CLGS1, CLGS2, and CLGS3 contained 19.6 ± 0.1, 20.3 ± 0.1, and 22.1 ± 0.1%, respectively. The CC, on the other hand, had a protein content of 19.1 ± 0.1%. Significant differences (*p* < 0.05) between cookies fortified with lupin sprouts and the control cookies were observed for all samples except the 10% addition of green sprouts in the cookie formula. However, no significant differences (*p* < 0.05) were observed in the lipid content of cookies with different percentages of lupin sprout flour, regardless of the added percentage. The analysis of these different lipid results led to several conclusions. Firstly, lupin improves the lipid composition of the cookies produced and this can be explained by the fact that lupin is richer in lipids than wheat flour [18,43,49]. This means lipid levels increase as the amount of lupin in the various cookies increases. Similar results were obtained by several researchers working on the use of lupin in bakery products [18,43,45]. Adding 20% lupin flour to wheat flour to create bread led to an increase in the lipid content from 0,95% in the control sample to 5.82% [43].

The carbohydrate composition of the CC was slightly higher (65.2%) than that of cookies with LGS (56.3–63.7%) and LFS (57.0–61.1%). This suggests that wheat flour provides more carbohydrates than lupin. In other studies, the carbohydrate content of composite flour with lupin varied between a value of 45.79 g/100 g for lupin flour and 80.10 g/100 g for wheat flour [18].

An important nutritional aspect is expressed by the calculation of the contribution that the consumption of 100 g of the product provides to the body in terms of the daily nutrient requirements. In this regard, the daily intakes (DIs) of the composite cookies, according Formulas (1)–(3) were determined. It can be observed (Table 2) that the protein intake for the daily consumption of 100 g of CLGS or CLSF cookies is higher than in the case of the CC, regardless of the percentage of sprouts added. The highest contribution to the daily protein intake was provided in the case of the consumption of 100 g of CLSF (18.104–26.598%), depending on the percentage of sprouts added, compared to 15.822% in the CC.

The consumption of cookies with lupin sprouts provided an important daily lipid intake compared to the daily requirement: in the range of 31.6–32.0% in the case of CLSF and 28.1–31.6% for CLGS, compared to 27.32% provided by the consumption of the CC. The carbohydrate daily intake was lower in the case of the consumption of cookies with lupin sprouts (21.6–24.5%) compared to the control (25.0%), but the contribution to the required daily energy value was higher.

To determine the mineral potential of different lupin cookies, the macro- and microelement compositions were determined (Table 3).

The results regarding the macro- and microelement compositions (Table 3) show an increase in nutrients with the addition of lupin sprouts to the cookie recipe. The higher macro- and microelement contents of the fortified cookies compared to the control are presented by the intake of by the lupin sprouts, either raw or dried. By drying, the contents of macro- and microelements in the plant matrix increase due to a decrease in the total plant mass by water release. The increases by drying the K content in LSF compared to LGS were 11.82% for Cu, 76.30% for Ni, 69.37% for Fe, and 215.87% for Mn. Significant increases were also observed for the macroelements: 50.35% for K, 59.64% for Ca, and 385.49% for Ca.

The analysis of the results obtained revealed that potassium (K) was the most abundant macroelement both in the control sample and in the various cookies, whether with lupin sprout flour (LSF) or lupin green sprout (LGS). The maximum value was recorded for the CLSF3 sample (544.07 mg/kg), with higher thanin in the CC and LGS cookies. The level of K increased with the addition of lupin sprouts to the cookie recipe. This finding corresponds with the results obtained by other authors, who reported the enrichment of the macroelemental composition of bakery products by fortifications with lentil flour and lentil sprouts [18,45,51]. The WHO (World Health Organization) guidelines for adults and children suggest that the potassium (K) intake should be at least 3510 mg/day for adults [52]. From this information, we deduced that consuming 100 g of CLSF3 daily covered 1.55% of daily K intake requirements. The calcium (Ca) content increased with the amount of lupin in the LSF and LGS cookies. This was 402.96 ± 0.16, 428.93 ± 0.18, and 463.09 ± 0.19 mg/kg for CLSF1, CLSF2, and CLSF3, and 87.73 ± 0.97, 109.9 ± 0.99, and 111.47 ± 0.75 mg/kg for CLGS1, CLGS2, and CLGS3, respectively. Sprouting increased the mineral contents. It was demonstrated that the calcium content of sprouted flour increased from 13.8 mg/100 g in non-sprouted red kidney beans to 16.1 mg/100 g in the sprouted flour [11].

The same observation was determined concerning magnesium (Mg) content. Increasing the quantity of lupin in the cookie recipe increased the Mg content of cookies with lupin sprouts. From 205.55 ± 0.20 mg/kg in CLSF1, the magnesium content increased to 248.10 ± 0.22 mg/kg in CLSF3, whereas from 76.54 ± 0.72 mg/kg in CLGS1, it increased by 4.07 mg/kg in CLGS3 (80.61 ± 0.8 mg/kg). For Ca and Mg contents, the cookies with LSF in the present study presented the same behavior as the bread produced in other studies: the higher the lupin content of the products, the higher the Ca and Mg contents [18,45]. According to the EFSA Scientific Panel, the recommended daily calcium intake for adults, including pregnant and lactating women, is no more than 2500 mg/day. For magnesium, however, the recommended intake is 350 mg/day for men and 300 mg/day for women. The recommended daily intake for children varies from 170 to 300 mg/day, depending on age. In our study, the consumption of 100 g of CLSF3 covered 1.85% of the daily Ca requirements for men and women, and between 8.27 and 14.59% of the daily Mg requirements for men, women, and children. CLGS3’s consumption (100 g) provides 0.35% of the daily Ca requirements for men and women. It also provides 2.19%, 2.55%, and 4.5% of the daily Mg requirements for men, women, and children, respectively. It can be concluded that cookies produced with LSF provide the body with more Mg and Ca than cookies produced with LGS.

In our study, cookies containing LSF were richer in iron and zinc than cookies containing LGS. A comparison between CLSF3 and CLGS3 shows that CLSF3 is approximately two- and three-times richer in Fe and Zn, respectively, than CLGS3.

Regarding the copper (Cu) content, an analysis of the data from this study showed that cookies with LSF were richer than cookies with LGS. On the other hand, studies show that the copper content of LSF cookies increases when lupin is abundant. Manganese (Mn) and nickel (Ni) behave the same as copper.

Previous studies confirm that germination increases the mineral content compared to non-germinated seeds and grains. The explanation may be due to the breakdown of phytates that act as chelators by the action of the enzyme phytase [17] or due to the enzymes that are liberated during seed germination, which break down complex structures to release minerals [11]. The sprouting period influences the level of elements. Ertas et al., (2015) recommend 3 days of germination for lupin flour obtained from sprouts for the nutritional enrichment of bread and for improving the technological quality with a minimum adverse effect on bread color [51].

Compared to the elemental content of ungerminated lupin flour [18], germination increases the macroelement intake in both LSF and LGS, resulting in a significant increase in minerals in fortified cookies. The increases in the macro- and microelement contents by the germination of lupin seeds were 374.13% for Ca, 344.60% for Mg, 741.48% for Mn, 90.09% for Fe, 145.48% for Zn, and 247.39% for Cu.

### 3.2. Phytochemical Profile of Composite Cookies

The phytochemical composition of the various lupin and control cookies was determined. The results of these various analyses are presented in Table 4.

Germination increases the antioxidant capacity in sprouts compared to the seeds from which they come. The value obtained for LSF (1081.37 µg Fe^2+/^g) was higher than the content in GLS (2511.62 µg Fe^2+/^g), while the drying process led to a concentration of the active principle relative to the weight of the dry material.

Relative to the value of flour obtained from ungerminated lupin seeds, it was observed that the TPC concentration increased from 128.65 mg/100 g to 828.510 mg/100 g in GLS and to 1512.281 mg/100 g in LSF.

Our study found that cookies produced with LSF were richer in total polyphenols than those produced with LGS. This was due to the fact that, by drying green sprouts, a greater number of phytonutrients was concentrated in the same percentage (10–30%).

An analysis of the total polyphenol content shows that different cookies with lupin are richer in total polyphenols than the control cookie and the level of TPC increases with the percentage of lupin sprouts added. The TPC ranged from 273.150 ± 1.01 to 546.330 ± 0.02 mg/100 g for CLSF cookies, and from 63.040 ± 0.05 to 79.485 ± 0.02 mg/100 g for CLGS cookies. Similar observations regarding the improvement of food functionality by adding lupin four were highlighted by other authors [18,49,53,54]. The TPC of lupin seeds varied in the range of 4260–5663 mg GAE/kg [55] and between 1.14–6.54 mgGAE/g, depending on the variety [56].

Germination has been shown to be a good process to increase the phenolic content of legume seeds and their antioxidant activity [21,23]. Also, the use of germinated seeds in the bakery industry improves the functionality of products. The phenolic content of biscuits containing 7.5% wheat sprouts resulted in the TPC increasing from 110 mg GAE/100 g DM in the control to 245 mg GAE/100 g DM in fortified biscuits [57].

On the other hand, according to the results of Yaver and Bilgiçli (2021), as the amount of lupin increased, the total polyphenol content decreased [45]. There are many possible reasons for this finding. The most plausible reason is the treatment of the lupin seeds. It was reported that the concentration of bound phenolic compounds depended on the release rate and conjugation at different stages of germination [17]. It was reported that soluble phenolic compounds could gradually accumulate in sprouted seeds based on the synthesis and transformation of new products [4,17]. The main precursor for phenol synthesis is glucose, so the high glucose concentration in sprouts can be related to the increase in polyphenols [58].

Our results show that the antioxidant activity (DPPH) of the CC (1.32 ± 0.03 µg/mL) is lower than that of other cookies with different proportions of lupin, whether with LSF or LGS. The values were 19.41 ± 0.38 µg/mL, 34.37 ± 1.20 µg/mL, and 44.89 ± 1.57 µg/mL for CLSF1, CLSF2 and CLSF3, respectively. For the CLGS1, CLGS2, and CLGS3 samples, the antioxidant values were 0.70 ± 0.02 µg/mL, 0.31 ± 0.01 µg/mL, 1.28 ± 0.01% µg/mL, 1.61 ± 0.01% µg/mL, and 1.77 ± 0.06 µg/mL. These results show increased antioxidant activity with the increased lupin sprout percentage in the cookies.

The values of antioxidant activity determined by the FRAP method presented the same profile as the total polyphenol content and antioxidant activity determined by the DPPH method. The CC had a lower antioxidant capacity (164.16 ± 1.52 Fe^2+^µg/g) than the two types of cookies with different proportions of lupin sprouts. The maximum value was recorded for CLSF3 (2433.52 ± 19.76 Fe^2+^µg/g); 316.16 ± 1.52 Fe^2+^µg/g was recorded for CLSG3. The increase was due to biologically active compounds in GLS (1081.37 ± 21.22 Fe^2+^µg/g) and germinated lupin flour (2511.62 ± 3.65 Fe^2+^µg/g).

The increase in the antioxidant capacity of cookies with composite lupin sprout flours as a functional ingredient was attributed to the antioxidant potential of lupin seeds [59,60,61]. Previous studies reported the antioxidant activity of lupin using DPPH and FRAP methods in the ranges of 0.99–1.87%, 5.49–7.92%, and 1.32–1.74 μmol TE/g [54]. The DPPH radical scavenging activity for lupin seeds of different varieties ranged from 0.39 to 3.50 mg TE/g and between 4.11 and 5.75 mg TE/g or 7.20 to 8.95 mg TE/g, when the CUPRAC method was used [55]. For all three phytochemical characteristics analyzed, there were statistically significant differences (*p* < 0.05) between the CC and all samples of cookies with lupin sprouts and between the CC, CLGS2, and CLGS3. Also, statistically significant differences (*p* < 0.05) between the two categories of cookies were observed. For the samples of cookies with lupin sprout flour, there were significant differences between the three variants for all the characteristics analyzed. In the case of the samples of cookies with lupin green sprout flour, except for polyphenols, there were significant differences between the three variants analyzed for the other characteristics.

### 3.3. Individual Polyphenols in Lupin Sprouts Cookies

The quantifications of some individual phenolic compounds in the cookies are shown in Table 5.

The individual polyphenols identified were epicatechin, caffeic acid, rutin, rosmarinic acid, quercetin, gallic, coumaric, ferulic, and beta-resorcylic. Protection against oxidative stress can be associated with these compounds’ powerful antioxidant and free radical scavenging properties [59].

The contents of caffeic acid and ferulic acid in the CC (17.4 ± 0.5 and 125.1 ± 9 µg/g, respectively) were lower than those of cookies with different percentages of lupin sprouts. Ferulic acid is a powerful antioxidant found in the cell walls of plants, particularly vegetables, cereals, and fruits [60]. Rosmarinic acid, quercetin, and gallic content had the same trend as caffeic and ferulic acids. On the other hand, values above the minimum detection limits were recorded for rutin either for the CC or for cookies with LSF (CLSF1, CLSF2, and CLSF3), and no significant differences for rutin content in cookies with LGS (CLGS1, CLGS2, and CLGS3) were observed. This suggests that transforming green sprouts into flour degrade its rutin content. This can be explained by the fact that lupin processing influences the phytochemical profile [61]. Other authors reported an important increase in rutin during the germination process. Thus, in green buckwheat sprouts, the rutin content increased to 109.0 mg/100 g after six days of germination, from 9.4 mg/100 g DW during pre-germination, approximately 1.5-times higher than the rutin content in non-germinated seeds [28]. Our results highlight significant differences between the rutin content in green sprouts and dry sprout flour. Koyama et al., (2013) reported that after soaking, the rutin content increased in the sprouts [28].

Regarding the epicatechin content, no significant differences were observed between the control sample and the cookies with lupin sprout flour (LSF). This suggests that the partial replacement of wheat flour with lupin sprout flour has no impact on the epicatechin composition of the cookies produced. On the other hand, although there was no significant difference between the values obtained for cookies produced with lupin green sprout (LGS), their values were slightly higher than those of the CC. The values were 74.2 ± 2, 73.9 ± 1.5, and 73.9 ± 1.2 µg/g for CLGS1, CLGS2, and CLGS3, respectively.

In the study of Vollmannova, A. et al., (2021) [55], individual phenolic contents (4-hydroxybenzoic acid, caffeic acid, trans-p-coumaric acid, trans-ferulic acid, myricetin, quercetin, apigenin, and genistein) from different lupin seeds varieties were determined. Caffeic acid (442.9–766.2 mg/kg) and myricetin (11.2–21.2 mg/kg) were the dominant phenolics in the investigated lupin cultivars [55]. Other authors reported that the dominant phenolics of all the lupin genotypes analyzed were p-coumaric acid derivatives (0.66–1.63 mg/g d.m.) and apigenin-6,8-di-C-glucoside (1.13–1.31 mg/g d.m.) [54]. Do Tan Khang et al. (2016) analyzed the phenolic profiles and antioxidant activity of germinated legumes and reported the values for coumaric acid in the range of 18.1–288.7 µg/g, 18.2–330.3 µg/g for ferulic acid, and 21.3 µg/g for gallic acid [21].

Germination produced significant changes in the flavonoids and non-flavonoid phenolic compounds [23]. Duenas et al., (2009) indicated that germination modified the quantitative and qualitative polyphenolic compositions of lupin *(Lupinus angustifolius* L.) seeds during the different days of germination. An increase in antioxidant activity was also observed due to the process [23].

### 3.4. Physical Evaluation of Lupin Sprout Cookies

The diameter, thickness, and spread ratio of lupin sprout cookies are presented in Table 6.

The diameter and the thickness decreased with the addition of LSF (Table 6). As the amount of lupin flour increased in the cookie recipe, its moisture and the thickness of the product decreased. It can be seen that the lower the water content, the smaller the thickness of the sample. Thus, we can deduce that, in order to obtain thicker cakes, we must use a softer dough, explained by the changes experience by the gluten [62]. The opposite pattern was observed for CLGS where the diameter increased with the addition of LGS to the cookie recipe, due to the increase in water content.

### 3.5. Sensory Property of Lupin Sprout Cookies

**Figure 5:**
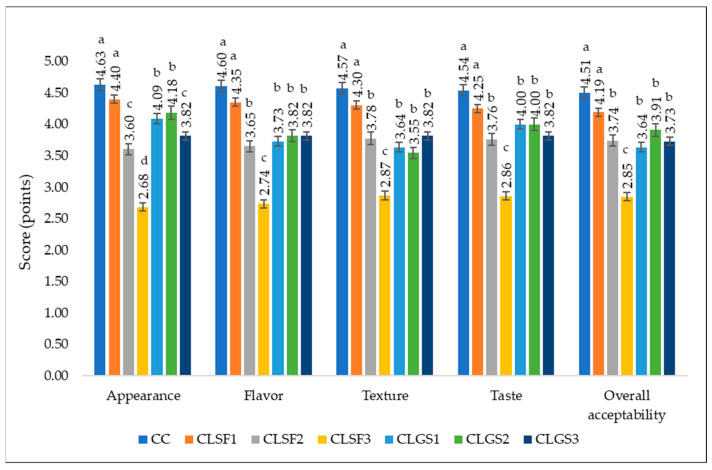
Sensory analysis of cookies using 5-point hedonic scale (n = 15). Values shown in the table represent the means of three independent results ± standard deviations (SDs). Data with different superscripts between columns for the same characteristic represent statistically significant differences according to one-way ANOVA (*p* < 0.05).

A sensory evaluation was conducted to determine how consumers liked the different cookies. This sensory evaluation covered the following attributes: appearance, aroma, texture, taste, and overall acceptability of the different cookies. The average score values obtained for the sensory analysis are shown in Figure 5. The results show that the control cookie obtained the highest score for all the analyzed attributes, followed by CLSF1, CLGS1, and CLSF2. CLSF3 and CLGS3 scored the most points for the flavor profile (neither like nor dislike) and poor taste, respectively.

The analysis of the various data from Figure 5 shows that the CC has the best scores, regardless of the sensory attributes studied in the present study. It scored 4.87 ± 0.35; 4.8 ± 0.56; 4.67 ± 0.62; 4.67 ± 0.72; and 4.73 ± 0.59 for appearance, flavor, texture, taste, and overall acceptability, respectively. All these values were between 4.5 and 5, so consumers highly appreciated the CC. Similar results were obtained by Kefale and Yetenayet (2020) [63]. These authors reported that the control sample of bread with 100% wheat flour was the most appreciated, regardless of the evaluation criteria. Another study highlighted that only in terms of the texture did the control bread sample obtain the highest score [18]. The differences observed in the results can be explained by the different treatments administered to the lupin, the part of the plant used (leaf, sprout, or seed), and the variety of lupin used.

Among the cookies with different proportions of lupin, CLSF1 scored the best for all sensory attributes. Its scores were close to those of the CC. Ranging from 3.5 to 5 for all sensory attributes, CLSF1 was highly appreciated for its appearance and slightly appreciated for all the other attributes. Comparing the scores of cookies with LSF (CLSF1, CLSF2, and CLSF3), we found that the more lupin there was in the cookies, the less consumers appreciated them. These results are similar to those reported in other studies regarding lupin-fortified products’ appearance, taste, texture, and overall acceptability [18,63]. These findings suggest that high levels of lupin reduce the consumer acceptance of cookies. Generally, bread produced with a maximum substitution of 20% germinated lupin flour does not significantly influence the sensory profile of the bread samples [26].

The same phenomenon was observed regarding the cookies with green sprout (LGS), but only for the texture, taste, and overall acceptability. Indeed, panel members were more likely to like CLGS2 than the other samples with LGS for its appearance and flavor. This meant that, in addition to the appearance and flavor, CLGS1 was the most appreciated cookie with LGS. Given that, in general, CLSF1 was the most appreciated, we deduced that the ideal level of substitution of wheat flour with lupin was 10% because, beyond 10%, consumers began to like the cookies less and less.

Other authors reported the influence of increasing the germination time in order to reduce the bitter taste. In this case, the fortification of bakery products enriched with lupin sprout flour can be increased up to 30% or even to 40 or 50% [64].

### 3.6. Glycemic Index (GI) of Lupin Sprout Cookies

Taking into account the results obtained regarding the nutritional analysis, the contribution to the required daily intake of nutrients, phytochemical profile, and sensory analysis, the 20% sample (CSFL2) was chosen for the study of the glycemic index.

**Figure 6:**
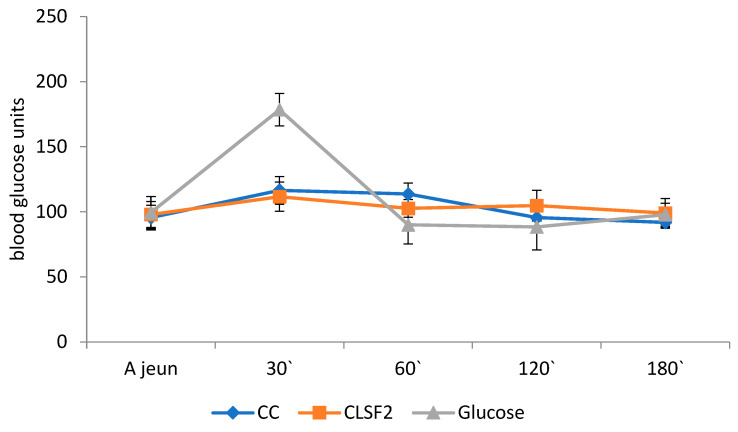
The comparison of postprandial blood glucose response within 120 min between glucose, control cookies (CCs), and cookies with 20% lupin sprout flour (CLSF2).

The experimental results for the evolution of blood glucose after the ingestion of 50 g of glucose and test products (CC and CSFL2) in an equivalent amount of 50 g of carbohydrates are shown in Figure 6.

The glycemic index study was conducted to observe the effect of incorporating the functional premix based on lupin sprout flour in a proportion of 20% on the postprandial blood glucose levels determined for the participants in this study. Figure 6 illustrates the average blood glucose levels at various intervals (baseline, 30, 60, 120, and 180 min) after the ingestion of 50 g of glucose (gray), control cookies (CCs) made from wheat flour (blue), and biscuits with 30% lupin sprout flour (CLSF2) (red) by 11 (8 women and 3 men) study participants with a normal body mass index (BMI).

Figure 6 shows that the baseline for blood glucose levels increases substantially for all participants after 30 min of pure glucose consumption. A more moderate increase was recorded in the cases of the CC and CLSF2. The functional food was distinguished by the lowest increase rate in blood glucose after 30 min compared to the baseline. Also, for most participants, there was no major difference in blood glucose levels at the five sampling times for this product.

The comparative graphic presentation of the blood glucose level obtained as the average of the blood glucose levels of the 11 participants in the study for the three cases tested (glucose, classic, and functional cookies) at five different analysis times (Figure 6) highlights the fact that, initially, at time zero, before ingestion, the blood sugar level was the same (around 100 units), on each of the 3 consecutive testing days. After 30 min from the ingestion of 50 g of glucose, i.e., produced in an amount equivalent to 50 g of carbohydrates, a moderate slope increase was observed, registering a maximum of 178.45 units in the case of the ingestion of pure glucose and lower values in the case of the control cookie (116.50 units), 111.63 in the case of the consumption of cookies with lupin sprout flour.

The blood sugar level showed a drastic decrease between 30 and 60 min to a value of 90.00 units, followed by a moderate decrease in the next 60 min to a value of 88.36 units, and it increased slightly to 97.72 units at the final moment of monitoring, in the case of pure glucose administration. Comparatively, for the tested products, there was a gradual decrease in the blood glucose level from 30 min to the end of the test interval, the decrease being much slower in the case of functional cookies compared to the classic ones, confirming the hypoglycemic potential of functional biscuits.

Carbohydrates are normally broken down into glucose once the test food has been ingested. The blood glucose level rises sharply to reach the highest concentration within 30 min and returns to the normal glucose level after 2 h, depending on the type of food consumed. The presence of fiber-rich ingredients interferes with digestion and delays the emptying of the gastrointestinal tract, affecting the absorption of nutrients, especially glucose [31].

**Figure 7:**
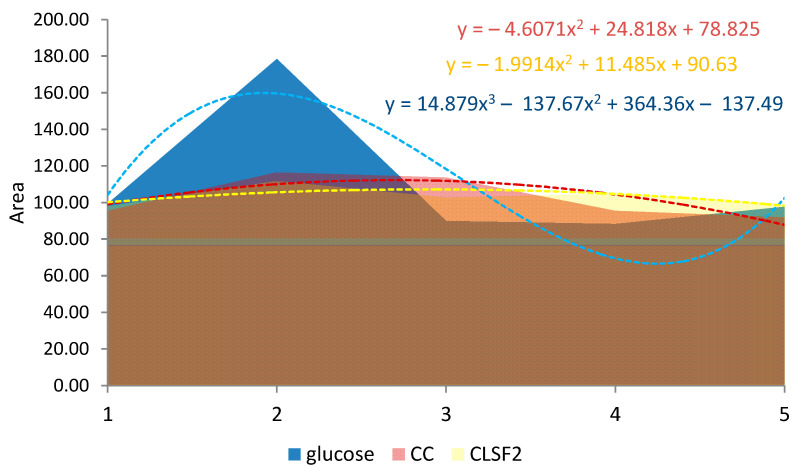
The glycemic response is calculated based on the incremental area under the curve (IAUC).

The glycemic response was further calculated to obtain each respondent’s incremental area under the curve (IAUC), which reflected changes in blood glucose levels over three hours (Figure 7). By calculating the ratio of the incremental areas under the curve (IAUC) for the tested products and glucose, according to equation (4), the glycemic index (GI) of the fortified biscuits with the addition of a functional premix based on lupin germ flour was determined compared to the glycemic index of some control biscuits produced with wheat flour. The GI value thus calculated for the control cookies (CCs) containing 50 g of carbohydrates was 78.196, while the GI for the cookies fortified with the functional premix based on lupin sprout flour 20% (CSFL2) was 75.654, lower than the control cookies.

Meta-analyses demonstrated that low-GI diets significantly improved glycemic control [65]. Germination is an important process in this control because it facilitates the enzymatic breakdown of carbohydrates into simple sugars by activating endogenous enzymes, such as α-amylase, thereby improving digestibility [66].

Further studies on sugar-free cookie recipes are necessary to justify the hypoglycemic character of biscuits with the addition of lupin sprouts.

## 4. Conclusions

Enriching cookies with different proportions of lupin sprout flour and lupin green sprouts significantly increased the nutritional composition of the resulting cookies, particularly in terms of the minerals, lipids, and proteins. It also improved the cookies’ composition regarding the total polyphenols, individual polyphenols, total flavonoids, and, finally, antioxidant activity. Regarding the sensory analysis, the adequate and acceptable level of substitution for consumers was 10%. Overall, for all the parameters examined in this study, although both types of cookies improved the cookies’ phytochemical, nutritional, and sensory properties, those with lupin sprout flour were the richest. Lupin sprout flour is thus a unique ingredient that provides better phytochemical and nutritional properties when enriching bakery products at substitution levels of between 10 and 30%. This study thus contributes to solution approaches of using natural and vegetable ingredients to improve the nutritional quality of products in the bakery sub-sector.

## Figures and Tables

**Table 1:** Recipe for control cookie (CC) and cookies with lupin sprout flour (CLSF) and lupin green sprout (LGS).

Samples	Ingredients						
	Lupin Sprout Flour (g)	Lupin Green Sprout (g)	Wheat Flour-Type 650 (g)	Baking Powder (g)	Butter 82% (g)	Eggs (pcs)	Sugar (g)
CC	-	-	100	1.4	50	1	34
CLSF1	10	-	90	1.4	50	1	34
CLSF2	20	-	80	1.4	50	1	34
CLSF3	30	-	70	1.4	50	1	34
CLGS1	-	10	90	1.4	50	1	34
CLGS2	-	20	80	1.4	50	1	34
CLGS3	-	30	70	1.4	50	1	34

**Table 2:** Proximate nutritional composition and contribution to the daily intake of lupin sprout cookies.

Nutritional Characteristics								
Samples	Moisture (%)	Mineral Content (%)	Proteins (%)	Dis ^1^ Protein (%)	Lipids (%)	Dis Lipid ^2^ (%)	Carbo-Hydrates (%)	Dis Carbo- Hydrates ^3^ (%)
LGS	58.5 ± 0.4	0.98 ± 0.10	33.9 ± 0.17	87.9	1.0 ± 0.1	1.4	8.5	3.2
LSF	10.2 ± 0.4	5.7 ± 0.21	28.2 ± 0.21	56.4	5.5 ± 0.3	7.9	50.2	19.3
CC	6.7 ± 0.2 ^d^	1.0 ± 0.1 ^a^	7.91 ± 0.13 ^e^	15.8	19.1 ± 0.1 ^d^	27.3	65.2	25.1
Cookies with Lupin Sprout Flour								
CLSF1	6.7 ± 0.2 ^d^	1.0 ± 0.1 ^a^	9.0 ± 0.1 ^c^	18.1	22.1 ±0.2 ^a^	31.6	61.1	23.5
CLSF2	6.1± 0.1 ^a^	1.3 ± 0.0 ^a^	12.1± 0.1^7 b^	24.3	22.3± 0.2 ^a^	31.9	57.9	22.3
CLSF3	5.8± 0.2 ^e^	1.3 ± 0.0 ^a^	13.3 ± 0.1 ^a^	26.6	22.4± 0.15 ^a^	32.0	57.0	21.9
Cookies with Lupin Green Sprouts								
CLGS1	7.5± 0.4 ^c^	1.1 ± 0.1 ^a^	7.9± 0.1 ^e^	15.9	19.6 ± 0.1 ^c^	28.1	63.7	24.5
CLGS2	9.3 ± 0.5 ^b^	1.3 ± 0.1 ^a^	8.4± 0.1 ^d^	16.9	20.3± 0.1 ^b^	29.1	60.6	23.3
CLGS3	11.3 ± 0.4 ^a^	1.5± 0.1 ^a^	8.6± 0.1 ^d^	17.2	22.1± 0.1 ^a^	31.6	56.3	21.6

^1–3^ Calculated according to Equations (1)–(3). Values shown in the table represent the means of three independent results ± standard deviations (SDs). Data with different superscripts in the same column represent statistically significant differences (one-way ANOVA, *p* < 0.05).

**Table 3:** Macro- and microelement contents of lupin sprout cookies.

Samples	Macro- and Microelement Contents (mg/kg)							
	Cu	Ni	Mn	K	Ca	Mg	Fe	Zn
LGS	10.75 ± 0.21	2.80 ± 0.22	364.85 ± 0.180	1249.44 ± 0.22	2195.23 ± 0.22	718.83 ± 0.17	15.52 ± 0.19	18.83 ± 0.16
LSF	12.02 ± 0.21	4.94 ± 0.19	1150.64 ± 0.21	1878.72 ± 0.20	1375.03 ± 0.17	3482.71 ± 0.19	41.82 ± 0.18	57.32 ± 0.19
CC	1.81 ± 0.2 ^c^	1.05 ± 0.17 ^c^	3.26 ± 0.18 ^f^	563.08 ± 0.19 ^c^	332.88 ± 0.15 ^d^	164.57 ± 0.20 ^d^	8.44 ± 0.18 ^c^	6.65 ± 0.21 ^c^
Cookies with Lupin Sprout Flour								
CLSF1	3.17 ± 0.19 ^b^	1.15 ± 0.19 ^c^	69.97 ± 0.20 ^c^	583.40 ± 0.1 ^b^	402.96 ± 0.16 ^c^	205.55 ± 0.20 ^c^	10.15 ± 0.20 ^b^	10.17 ± 0.19 ^b^
CLSF2	3.94 ± 0.17 ^a^	1.74 ± 0.14 ^b^	170.52 ± 0.16 ^b^	613.15 ± 0.20 ^a^	428.93 ± 0.18 ^b^	212.75 ± 0.21 ^b^	11.12 ± 0.19 ^b^	12.54 ± 0.19 ^a^
CLSF3	4.14 ± 0.18 ^a^	2.02 ± 0.18 ^a^	242.70 ± 0.20 ^a^	544.07 ± 0.17 ^d^	463.09 ± 0.19 ^a^	248.10 ± 0.22 ^a^	14.70 ± 0.20 ^a^	12.94 ± 0.18 ^a^
Cookies with Lupin Green Sprout								
CLGS1	0.97 ± 0.148 ^e^	0.24 ± 0.13 ^e^	7.13 ± 0.81 ^e^	303.27 ± 0.81 ^f^	87.73 ± 0.97 ^f^	76.54 ± 0.72 ^f^	8.15 ± 0.82 ^c^	3.82 ± 0.88 ^e^
CLGS2	0.99 ± 0.165 ^e^	0.38 ± 0.1 ^d^	19.44 ± 0.85 ^d^	329.48 ± 0.87 ^e^	109.9 ± 0.99 ^e^	79.14 ± 0.86 ^e^	8.21 ± 0.86 ^c^	5.75 ± 0.84 ^d^
CLGS3	1.25 ± 0.183 ^d^	0.45 ± 0.12 ^d^	19.61 ± 0.88 ^d^	330.73 ± 0.90 ^e^	111.47 ± 0.75 ^e^	80.61 ± 0.80 ^e^	8.64 ± 0.8 ^c^	6.13 ± 0.81 ^c^

Values shown in the table represent the means of three independent results ± standard deviations (SDs). Data with different superscripts in the same column represent statistically significant differences (one-way ANOVA, *p* < 0.05).

**Table 4:** Phytochemical composition of lupin sprout cookies.

Samples	Total Polyphenol Content (TPC) (mg/100 g)	Antioxidant Activity, DPPH (µgTROLOX/mL)	FRAP (µg Fe^2+^/g)
LGS	828.51 ± 0.01	104.57 ± 1.04	1081.37 ± 21.22
LSF	1512.28 ± 0.25	189.75 ± 1.61	2511.62 ± 3.65
CC	51.86 ± 0.01 ^g^	1.32 ± 0.03 ^e^	164.16 ± 1.52 ^g^
Cookies with Lupin Sprout Flour			
CLSF1	273.15 ± 1.01 ^c^	19.41 ± 0.38 ^c^	1182.56 ± 6.08 ^c^
CLSF2	436.45 ± 1.01 ^b^	34.37 ± 1.20 ^b^	1916.72 ± 22.8 ^b^
CLSF3	546.33 ± 0.02 ^a^	44.89 ± 1.57 ^a^	2433.52 ± 19.76 ^a^
Cookies with Lupin Green Sprout			
CLGS1	63.04 ± 0.05 ^f^	0.70 ± 0.02 ^f^	194.56 ± 1.52 ^f^
CLGS2	69.22 ± 0.05 ^e^	0.31 ± 0.01 ^g^	244.72 ± 1.52 ^e^
CLGS3	79.48 ± 0.02 ^d^	1.77 ± 0.06 ^d^	316.16 ± 1.52 ^d^

Values shown in the table represent the means of three independent results ± standard deviations (SDs). Data with different superscripts in the same column represent statistically significant differences (one-wayANOVA, *p* < 0.05).

**Table 5:** Quantification of individual polyphenols using LC-MS.

Samples	Individual Polyphenols (µg/g)								
	Epicatechin	Caffeic Acid	Rutin	Rosmarinic Acid	Quercetin	Gallic Acid	Coumaric Acid	Ferulic Acid	Beta- Resorcylic Acid
CC	73.8 ± 3.1 ^a^	17.4 ± 0.5 ^a^	Nd *	68.0 ± 1.7 ^a^	15.7 ± 3.1 ^a^	16.9 ± 0.7 ^b^	41.9 ± 1.8 ^b^	125.1 ± 9 ^a^	175.2 ± 9.5 ^a^
Cookies with Lupin Sprout Flour									
CLSF1	74.2 ± 3.1 ^a^	17.5 ± 2 ^a^	Nd *	68.1 ± 1.5 ^a^	15.8 ± 4.2 ^a^	17.1 ± 0.9 ^b^	40.9 ± 1.2 ^b^	125.4 ± 8.1 ^a^	175.3 ± 9.1 ^a^
CLSF2	74.8 ± 3.1 ^a^	17.7 ± 1.2 ^a^	Nd *	68.3 ± 5.1 ^a^	16.0 ± 3.5 ^a^	18.7 ± 0.8 ^a^	40.5 ± 1.7 ^b^	125.1 ± 7.8 ^a^	175.7 ± 8.5 ^a^
CLSF3	74.8 ± 3.1 ^a^	17.9 ± 2.7 ^a^	Nd *	69.1 ± 2.4 ^a^	16.0 ± 1.9 ^a^	18.7 ± 0.8 ^a^	41.6 ± 0.8 ^b^	126.1 ± 7.9 ^a^	176.4 ± 8.1 ^a^
Cookies with Lupin Green Sprout									
CLGS1	74.2 ± 2 ^a^	17.4 ± 0.2 ^a^	78.3 ± 7.5 ^a^	68.0 ± 1.7 ^a^	15.8 ± 2.9 ^a^	16.7 ± 0.7 ^b^	41.4 ± 1.5 ^b^	124.9 ± 6.8 ^a^	175 ± 7.5 ^a^
CLGS2	73.9 ± 1.5 ^a^	17.5 ± 0.4 ^a^	79.3 ± 7.5 ^a^	68.8 ± 0.5 ^a^	15.7 ± 2.3 ^a^	17.0 ± 1 ^b^	41.1 ± 1.5 ^b^	125.3 ± 7.5 ^a^	175.1 ± 6.1 ^a^
CLGS3	73.9 ± 1.2 ^a^	18.6 ± 0.4 ^a^	79.8 ± 7.5 ^a^	68.1 ± 1.3 ^a^	15.7 ± 0.9 ^a^	17.4 ± 0.5 ^b^	43 ± 1.6 ^a^	126.9 ± 7.1 ^a^	177.1 ± 7.6 ^a^

* Nd—not detectable. Values shown in the table represent the means of three independent results ± standard deviations (SDs). Data with different superscripts in the same column represent statistically significant differences (one-way ANOVA, *p* < 0.05).

**Table 6:** Thickness, diameter, and spread ratio of lupin sprout cookies.

Samples	Physical Characteristics		
	Diameter (mm)	Thickness (mm)	Spread Ratio
Cookies with Lupin Green Sprouts			
CC	49.00 ± 0.25 ^e^	5.25 ± 0.15 ^a^	
CLSF1	51.50 ± 1.42 ^c^	4.95 ± 0.20 ^b^	10.42 ^b^
CLSF2	50.80 ± 0.63 ^d^	4.80 ± 0.34 ^b^	10.94 ^b^
CLSF3		4.70 ± 0.10^,b^	11.23 ^a^
Cookies with Lupin Green Sprouts			
CLGS1	50.40 ± 0.38 ^d^	4.40 ± 1.42^,b^	11.45 ^a^
CLGS2	52.25 ± 0.75 ^b^	4.55 ± 1.42 ^b^	11.48 ^a^
CLGS3	54.55 ± 0.25 ^a^	4.65 ± 1.42 ^b^	11.73 ^a^

Values shown in the table represent the means of three independent results ± standard deviations (SDs). Data with different superscripts in the same column represent statistically significant differences (one-way ANOVA, *p* < 0.05).

## Data Availability

The report of the analyses performed for the samples in the paper can be found at the Interdisciplinary Research Platform (PCI) at the University of Life Sciences “King Mihai I”, Timisoara.
